# Common Genetic Variants in Wnt Signaling Pathway Genes as Potential Prognostic Biomarkers for Colorectal Cancer

**DOI:** 10.1371/journal.pone.0056196

**Published:** 2013-02-06

**Authors:** Wen-Chien Ting, Lu-Min Chen, Jiunn-Bey Pao, Ying-Pi Yang, Bang-Jau You, Ta-Yuan Chang, Yu-Hsuan Lan, Hong-Zin Lee, Bo-Ying Bao

**Affiliations:** 1 Department of Colorectal Surgery, China Medical University Hospital, Taichung, Taiwan; 2 Division of Colorectal Surgery, Department of Surgery, Chung Shan Medical University Hospital, Taichung, Taiwan; 3 Institute of Medicine, Chung Shan Medical University, Taichung, Taiwan; 4 Department of Obstetrics and Gynecology, China Medical University Hospital, Taichung, Taiwan; 5 Department of Pharmacy, Yangming Branch, Taipei City Hospital, Taipei, Taiwan; 6 Department of Chinese Medicine Resources, China Medical University, Taichung, Taiwan; 7 Department of Occupational Safety and Health, China Medical University, Taichung, Taiwan; 8 Department of Pharmacy, China Medical University, Taichung, Taiwan; 9 Sex Hormone Research Center, China Medical University Hospital, Taichung, Taiwan; National Cancer Center, Japan

## Abstract

Compelling evidence has implicated the Wnt signaling pathway in the pathogenesis of colorectal cancer. We assessed the use of tag single nucleotide polymorphisms (tSNPs) in *adenomatous polyposis coli* (*APC*)/*β-catenin* (*CTNNB1*) genes to predict outcomes in patients with colorectal cancer. We selected and genotyped 10 tSNP to predict common variants across entire *APC* and *CTNNB1* genes in 282 colorectal cancer patients. The associations of these tSNPs with distant metastasis-free survival and overall survival were evaluated by Kaplan-Meier analysis, Cox regression model, and survival tree analysis. The 5-year overall survival rate was 68.3%. Survival tree analysis identified a higher-order genetic interaction profile consisting of the *APC* rs565453, *CTNNB1* 2293303, and *APC* rs1816769 that was significantly associated with overall survival. The 5-year survival overall rates were 89.2%, 66.1%, and 58.8% for the low-, medium-, and high-risk genetic profiles, respectively (log-rank *P* = 0.001). After adjusting for possible confounders, including age, gender, carcinoembryonic antigen levels, tumor differentiation, stage, lymphovascular invasion, perineural invasion, and lymph node involvement, the genetic interaction profile remained significant. None of the studied SNPs were individually associated with distant metastasis-free survival and overall survival. Our results suggest that the genetic interaction profile among Wnt pathway SNPs might potentially increase the prognostic value in outcome prediction for colorectal cancer.

## Introduction

Colorectal cancer is a common disease worldwide, and is also the most common cancer in Taiwan. The 5-year survival rates are approximately 90% for early stage colorectal cancer patients, but decreasing to less than 10% in patients with distant metastases [1]. To identify patients at high risk, there is a need to find new biomarkers to improve the prediction of clinical outcomes in colorectal cancer.

Mutations in Wnt/adenomatous polyposis coli (APC)/β-catenin (CTNNB1) signaling pathway members have been found in many colorectal carcinomas [2]. Wnt ligands bind to transmembrane Frizzled receptors and their co-receptors, leading to phosphorylation and sequestration of the complex composed of APC, casein kinase 1, glycogen synthase kinase 3, and axin. The resultant stabilization of intracellular CTNNB1 facilitates its translocation to the nucleus, where it interacts with transcription factors of the T-cell factor/lymphoid enhancer-binding factor, activating the targets controlling cell growth and differentiation. Therefore, the interaction with CTNNB1 has been considered to be essential for the tumor suppressor activity of APC [3]. In addition, CTNNB1 is a multifunctional signaling protein, which also binds to E-cadherin, linking E-cadherin to actin filaments and promoting cell adhesion and differentiation [4].

In light of the critical role of the Wnt/APC/CTNNB1 signaling pathway in maintaining proper colorectal cell function, it is possible that genetic variants in this pathway might affect colorectal cancer progression. However, there have been no studies addressing the relation of common genetic variants in Wnt/APC/CTNNB1 pathway genes to clinical outcomes of colorectal cancer. Therefore, we applied a comprehensive approach to systematically evaluate the tag single-nucleotide polymorphisms (tSNPs) in two key genes in the Wnt pathway, *APC* and *CTNNB1*, as predictors of colorectal cancer prognosis.

## Patients and Methods

### Patient Recruitment and Data Collection

This cohort was generated from the tumor tissue bank at China Medical University Hospital, Taiwan. All patients seen at China Medical University Hospital, Taiwan with a diagnosis of cancers were approached to participate. Two hundred and eighty-two patients with histopathologically confirmed colorectal cancer were identified between 2001 and 2007; had consented to provide information and tissue; and had undergone blood collection for research purposes. The clinical data and outcomes were obtained from patients’ clinical records and pathological reports. Among patients receiving curative surgery (stage I-III, n = 233), distant metastasis-free survival was defined as the time from surgery to the date of distant metastases or when censored at the latest date. Overall survival was defined as the time from diagnosis (n = 282) to the date of death from any cause or when censored at the latest date if patients were still alive. The survival data were updated most recently in 2010. This study was approved by the Institutional Review Board of the China Medical University Hospital and written informed consent was obtained from all patients.

### SNP Selection and Genotyping

SNP selection has been previously described [5]. Briefly, we determined the genetic structure of *APC* and *CTNNB1* by using publicly available genotype data from the HapMap consortium [6]. According to HapMap CHB (Han Chinese in Beijing, China) population data, a total of 78 SNPs and 36 SNPs with a minor allele frequency (MAF) >0.10 spanned 20 kb of the 5′ upstream and 10 kb of the 3′ downstream of *APC* and *CTNNB1* genes, respectively (chromosome 5∶112081483- 112219834 for *APC*, chromosome 3∶41196016- 41266938 for *CTNNB1*). Using the Tagger algorithm [7] implemented in the HaploView program [8] and dense genotyping data from the HapMap CHB population, we identified 7 and 5 linkage disequilibrium tSNPs to capture unmeasured genetic variations with *r* ^2^ greater than 0.8 in *APC* and *CTNNB1*, respectively.

Genomic DNA was extracted from peripheral blood using the QIAamp DNA Blood Mini Kit (Qiagen, Valencia, CA, USA) and stored at -80°C until the time of study. Genotyping was performed as described previously [9] using Sequenom iPLEX matrix-assisted laser desorption/ionization-time of flight (MALDI-TOF) mass spectrometry technology at the National Center for Genome Medicine, Academia Sinica, Taiwan. The average genotype call rate for these SNPs was 98.9%. Any SNP that did not conform to Hardy-Weinberg equilibrium (*P*<0.001) was removed (n = 2). Thus, a total of 10 tSNPs were included for further statistical analyses.

### Statistical Analysis

Patient clinicopathologic characteristics were summarized as number and percentage of patients or median and interquartile range (IQR) of values. Age was dichotomized at the median value within the cohort. Carcinoembryonic antigen (CEA) level was dichotomized at 5 µg/L because of its correlation with an increasing stage of the colorectal cancer [10]. The associations of 10 individual SNPs and clinical characteristics with distant metastasis-free and overall survival were assessed using the Kaplan-Meier analysis with log-rank test. Higher order SNP-SNP interactions were evaluated using survival tree analysis by STREE software (http://c2s2.yale.edu/software/stree/), which uses recursive partitioning to identify subgroups of individuals with similar risk of death [11]. The tree structure starts with the root that includes all study population, and uses the log-rank statistic to select optimal split that classified patients into low and high risk groups. The recursive procedure continues to produce subsequent groups that are more homogeneous than the original group. The final model is a tree structure with many binary splits, and each terminal represents a subgroup of patients with different risks of death based on distinct genotype combinations. Kaplan-Meier analysis with log-rank test was then used to estimate the survivals between each of the terminal subgroups. Multivariate analyses to determine the interdependency of genotypes and other known prognostic factors, such as age at diagnosis, gender, CEA levels, tumor differentiation, stage, lymphovascular invasion, perineural invasion, and lymph node involvement, were carried out using Cox proportional hazards regression model. Statistical Package for the Social Sciences software version 19.0.0 (IBM, Armonk, NY) was used for other statistical analyses. A two-sided *P* value of <0.05 was considered statistically significant.

## Results

The demographic data of colorectal cancer patients are presented in Table 1. During the median follow-up of 54.5 months, 30 patients developed distant metastasis and the 2-year distant metastasis-free survival rate was 87.1%. Ninety-two patients died after a median follow-up of 50.0 months and the 5-year overall survival rate was 68.3%. Clinical variables significantly associated with both distant metastasis-free and overall survival included tumor differentiation, stage, lymphovascular invasion, and lymph node involvement. Age was only associated with distant metastasis-free survival, and gender, CEA levels, and perineural invasion were only associated with overall survival.

**Table 1:** Demographic and clinical characteristics of colorectal cancer patients.

Characteristics	Distant metastasis-free survival				Overall survival			
	n (%)*	n ofevents*	2-year survivalrate (%)	*P* †	n (%)*	n ofevents*	5-year survivalrate (%)	*P* †
All patients	233	30	87.1		282	92	68.3	
Age, years								
Median (IQR)	66 (56–73)				65 (54–73)			
<65	106 (45.5)	19	82.1	**0.044**	133 (47.2)	42	68.1	0.692
≥65	127 (54.5)	11	91.3		149 (52.8)	50	68.5	
Gender								
Male	119 (51.1)	17	85.7	0.507	148 (52.5)	57	62.5	**0.043**
Female	114 (48.9)	13	88.6		134 (47.5)	35	75.1	
CEA, µg/L								
Median (IQR)	3.5 (1.7–10.2)				4.4 (2.0–14.9)			
<5	129 (59.7)	14	89.1	0.194	136 (52.3)	23	83.5	**<0.001**
≥5	87 (40.3)	15	82.8		124 (47.7)	58	54.7	
Differentiation								
Well	43 (20.0)	5	88.4	**0.022**	51 (19.4)	11	78.1	**0.002**
Moderate	153 (70.8)	17	88.9		185 (70.3)	62	67.6	
Poor	19 (9.2)	6	68.4		27 (10.3)	15	44.4	
Stage‡								
I-II	150 (64.7)	12	92.0	**0.002**	–			
III	82 (35.3)	18	78.0		–			
I-III	–				232 (82.6)	54	77.3	**<0.001**
IV	–				49 (17.4)	38	25.3	
Lymphovascular invasion								
Negative	179 (76.8)	14	92.2	**<0.001**	201 (71.3)	48	77.4	**<0.001**
Positive	54 (23.2)	16	70.4		81 (28.7)	44	45.8	
Perineural invasion								
Negative	202 (86.7)	23	88.6	0.100	234 (83.0)	65	73.0	**<0.001**
Positive	31 (13.3)	7	77.4		48 (17.0)	27	45.7	
Lymph node involvement								
Negative	143 (64.1)	10	93.0	**0.002**	154 (56.8)	33	80.2	**<0.001**
Positive	80 (35.9)	17	78.8		117 (43.2)	56	52.3	

Abbreviations: CEA, carcinoembryonic antigen; IQR, interquartile range.

* Column subtotals do not sum to n of patients and n of events due to missing data.

† *P* values were calculated using the log-rank test.

‡ According to the American Joint Committee on Cancer - Cancer Staging Manual (version 6.0).

*P*<0.05 are in boldface.

**Figure 1:**
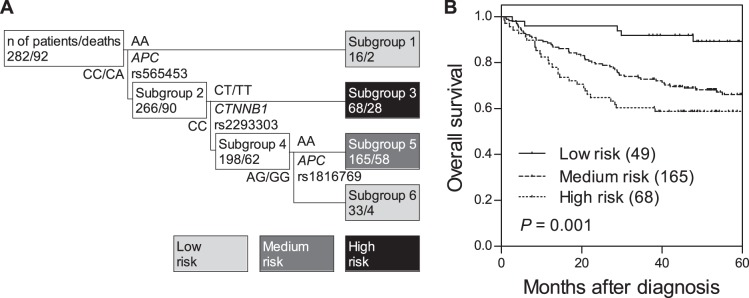
Potential higher order SNP-SNP interactions among Wnt pathway gene polymorphisms. (A) Survival tree analysis identifies the interactions among the three polymorphisms. (B) Kaplan-Meier curves of overall survival based on the survival tree analysis. Numbers in parentheses indicate the number of patients.

A total of 10 tSNPs within *APC* and *CTNNB1* were analyzed. We assessed the association of each individual SNP with survival status under dominant, recessive and additive models using the log-rank test (Table S1). We did not observe any noteworthy association of tSNPs in Wnt pathway genes with metastasis-free survival, overall survival, and clinical characteristics listed in Table 1 (data not shown). Therefore, we further explored higher order SNP-SNP interactions to evaluate whether the interactions among these tSNPs could determine the clinical outcomes. Survival tree analysis identified higher order genetic interactions among *APC* rs565453, *CTNNB1* 2293303, and *APC* rs1816769, and the final tree structure identified four terminal subgroups with low-, medium-, and high-risk of death according to the Kaplan-Meier analysis (Figure 1A). Since subgroups 1 and 6 had very few cases (16 and 33 patients, respectively) and had similar risks, we combined them as the low-risk group. The low-risk genetic profile group had a 5-year overall survival rate of 89.2%. In comparison, the medium-risk group had a 5-year overall survival rate of 66.1% and the hazard ratio (HR) was 3.25 [95% confidence interval (CI): 1.40–7.54, *P* = 0.006, Figure 1B and Table 2]. The high-risk group had a 5-year overall survival rate of 58.8% and the HR was 4.39 (95% CI: 1.82–10.6, *P* = 0.001). However, no genetic interaction was observed for distant metastasis-free survival.

**Table 2:** Cox proportional hazards analysis of factors associated with overall survival.

Variables	Univariate analysis		Multivariate analysis*	
	HR (95% CI)	*P*	HR (95% CI)	*P*
Age, years				
<65	1.00		1.00	
≥65	1.09 (0.72–1.64)	0.692	1.29 (0.78–2.14)	0.328
Gender				
Male	1.00		1.00	
Female	0.65 (0.43–0.99)	**0.045**	0.75 (0.46–1.22)	0.245
CEA, µg/L				
<5	1.00		1.00	
≥5	3.35 (2.07–5.44)	**<0.001**	1.92 (1.10–3.34)	**0.021**
Differentiation				
Well	1.00		1.00	
Moderate	1.64 (0.86–3.12)	0.131	1.66 (0.80–3.45)	0.171
Poor	3.59 (1.65–7.83)	**0.001**	4.25 (1.73–10.5)	**0.002**
Stage				
I–III	1.00		1.00	
IV	5.19 (3.40–7.92)	**<0.001**	3.61 (2.11–6.18)	**<0.001**
Lymphovascular invasion				
Negative	1.00		1.00	
Positive	2.78 (1.84–4.19)	**<0.001**	1.48 (0.79–2.77)	0.225
Perineural invasion				
Negative	1.00		1.00	
Positive	2.39 (1.53–3.75)	**<0.001**	1.20 (0.65–2.24)	0.561
Lymph node involvement				
Negative	1.00		1.00	
Positive	2.76 (1.79–4.24)	**<0.001**	1.64 (0.96–2.80)	0.073
Wnt pathway gene polymorphisms				
Low risk	1.00		1.00	
Medium risk	3.25 (1.40–7.54)	**0.006**	3.67 (1.43–9.44)	**0.007**
High risk	4.39 (1.82–10.6)	**0.001**	4.57 (1.73–12.1)	**0.002**

Abbreviations: CEA, carcinoembryonic antigen; HR, hazard ratio; CI, confidence interval.

* Age, gender, CEA levels, tumor differentiation, stage, lymphovascular invasion, perineural invasion, lymph node involvement, and genetic risk classification by Wnt pathway gene polymorphisms were included in the multivariate analysis.

*P*<0.05 are in boldface.

To assess the predictive effects of the genetic interaction profile beyond the clinical features to influence overall survival, we performed a multivariate analysis, adjusting for age, gender, CEA levels, tumor differentiation, stage, lymphovascular invasion, perineural invasion, and lymph node involvement. After controlling for these predictors, the genetic interaction remained significant. In comparison to the low-risk genetic profile group, the medium-risk group presented a 3.67-fold increased risk of death (adjusted HR: 3.67, 95% CI: 1.43–9.44, *P* = 0.007, Table 2), and the high-risk group had a 4.57-fold increased risk of death (adjusted HR: 4.57, 95% CI: 1.73–12.1, *P* = 0.002). These data suggest that the genetic interaction profile among Wnt pathway SNPs might be an independent outcome predictor for colorectal cancer.

## Discussion

This study evaluated the prognostic significance of common genetic variants in the Wnt/APC/CTNNB1 pathway genes on survival in colorectal cancer patients. A higher-order genetic interaction profile consisting of the *APC* rs565453, *CTNNB1* 2293303, and *APC* rs1816769 were associated with overall survival. Notably, the relationships between the genetic interaction profile and survival persisted despite controlling for known clinical prognostic factors.

APC is generally considered a key component of the Wnt signaling pathway, and its primary function as tumor suppressor is believed to be its ability to negatively regulate the Wnt pathway. rs565453 is located in the 3′ downstream of the *APC* gene, and is predicted to alter a putative transcription factor binding site for OCT1 transcription factor, according to the prediction of SNP Function Portal [12]. OCT1 expression is increased in several human cancers and has been suggested to be associated with the onset or progression of cancer or with the resistance of tumor cells to radiotherapy or chemotherapy [13]. Although *APC* rs1816769 in intron 4 has no predicted function, it locates in a putative mammalian transposable element, L1MC4a. The transposable elements in human genome are previously considered harmless, but now known to have impact on gene expression via modification of the transcript quality or quantity, transcriptional interference, or by the control of pathways that affect the mRNA life-cycle [14]. CTNNB1 is not only a key nuclear effector of Wnt signaling in the nucleus, but also a structural component of cadherin-based adherens junctions. Imbalance in the structural and signaling properties of CTNNB1 often results in deregulated growth connected to cancer and metastasis [15]. *CTNNB1* rs2293303 is a synonymous coding SNP, but the risk allele, T, is predicted to disrupt a putative exonic splicing enhancer motif responsive to the human SR protein SF2/ASF. Therefore, it is plausible that these SNPs might influence APC/CTNNB1 splicing and expression by altering the consensus splicing site sequences, the transposable elements, and the transcription factor binding sites. Further fine-mapping and functional analyses are required to identify the potential causative SNPs and to understand the roles of APC/CTNNB1 in colorectal cancer progression.

In summary, this might be the first study to systematically evaluate the use of tSNPs in *APC* and *CTNNB1* genes to predict outcomes in colorectal cancer patients. We found that a higher-order genetic interaction profile among Wnt pathway SNPs was associated with overall survival, even after adjusting for clinical predictors of outcome. The results reported here are limited by analyzing the small number of patients. In addition, our homogeneous Chinese Han population might make our findings less generalizable to other ethnic groups. Independent external studies are necessary to validate our findings. If validated, these biomarkers might be valuable to facilitate individualized treatment decisions.

## Acknowledgments

We thank National Genotyping Center of National Research Program for Genomic Medicine, National Science Council, Taiwan, for their technical support.

## Supporting Information

Table S1 **Genotyped polymorphisms and the** ***P*** **values of their association with distant metastasis-free survival and overall survival.**
(DOC)
