# Supplementary material for: Common Genetic Variants in Wnt Signaling Pathway Genes as Potential Prognostic Biomarkers for Colorectal Cancer
Source: PLoS One. 2013 Feb 6;8(2):e56196. doi: 10.1371/journal.pone.0056196 (PMC3566082; doi:10.1371/journal.pone.0056196)
Supplement: Table S1 — Genotyped polymorphisms and the P values of their association with distant metastasis-free survival and overall survival. (DOC) [file pone.0056196.s001.doc]

**Table S1.** Genotyped polymorphisms and the *P* values of their association with distant metastasis-free survival and overall survival

| Gene | tSNP ID | Position | Location | Allele | Log-rank *P* | | | | | |
| --- | --- | --- | --- | --- | --- | --- | --- | --- | --- | --- |
|  |  |  |  |  | Distant metastasis-free survival | | | Overall survival | | |
|  |  |  |  |  | Additive | Dominant | Recessive | Additive | Dominant | Recessive |
| *APC* | rs11950612 | Chr5:112114773 | Intron 1 | G>A | 0.827 | 0.808 | 0.252 | 0.674 | 0.944 | 0.318 |
|  | rs1816769 | Chr5:112137678 | Intron 4 | A>G | 0.060 | 0.061 | 0.324 | 0.146 | 0.135 | 0.529 |
|  | rs2289484 | Chr5:112144672 | Intron 6 | A>G | 0.152 | 0.125 | 0.469 | 0.082 | 0.148 | 0.148 |
|  | rs501250 | Chr5:112183692 | Intron 10 | A>G | 0.082 | 0.086 | 0.357 | 0.140 | 0.111 | 0.648 |
|  | rs565453 | Chr5:112213292 | 3' downstream | C>A | 0.176 | 0.165 | 0.446 | 0.111 | 0.249 | 0.080 |
| *CTNNB1* | rs1798802 | Chr3:41236983 | Intron 1 | G>A | 0.753 | 0.685 | 0.985 | 0.920 | 0.467 | 0.267 |
|  | rs3774369 | Chr3:41242529 | Intron 6 | A>G | 0.521 | 0.358 | 0.729 | 0.374 | 0.290 | 0.994 |
|  | rs4135385 | Chr3:41254444 | Intron 13 | G>A | 0.441 | 0.663 | 0.393 | 0.291 | 0.604 | 0.208 |
|  | rs11564475 | Chr3:41255037 | Intron 14 | A>G | 0.314 | 0.546 | 0.117 | 0.978 | 0.845 | 0.500 |
|  | rs2293303 | Chr3:41255831 | Asp780Asp | C>T | 0.267 | 0.141 | 0.454 | 0.318 | 0.156 | 0.427 |

Abbreviations: tSNP, tag single nucleotide polymorphism.

*P* < 0.05 are in boldface.
